# Identification of a Xist silencing domain by Tiling CRISPR

**DOI:** 10.1038/s41598-018-36750-0

**Published:** 2019-02-20

**Authors:** Yang Wang, Yang Zhong, Yingyao Zhou, Olga Tanaseichuk, Zhizhong Li, Jing Crystal Zhao

**Affiliations:** 1 Tumor Initiation and Maintenance Program, NCI-designated Cancer Center, Sanford Burnham Prebys Medical Discovery Institute, La Jolla, CA 92037 USA; 2 Genomics Institute of the Novartis Research Foundation, 10675 John Jay Hopkins Drive, San Diego, CA 92121 USA

## Abstract

Despite essential roles played by long noncoding RNAs (lncRNAs) in development and disease, methods to determine lncRNA *cis*-elements are lacking. Here, we developed a screening method named “Tiling CRISPR” to identify lncRNA functional domains. Using this approach, we identified Xist A-Repeats as the silencing domain, an observation in agreement with published work, suggesting Tiling CRISPR feasibility. Mechanistic analysis suggested a novel function for Xist A-repeats in promoting Xist transcription. Overall, our method allows mapping of lncRNA functional domains in an unbiased and potentially high-throughput manner to facilitate the understanding of lncRNA functions.

## Introduction

The realization that numerous lncRNAs likely function in disease initiation and progression has opened up unlimited possibilities in terms of novel therapies or diagnostics. However, although we can detect and quantify lncRNAs in biopsy tissues and cell lines, our knowledge of their molecular function remains a roadblock to developing lncRNAs as drug targets. Multiple functional mechanisms have been proposed for lncRNAs, including serving as a scaffold for assembly of protein complexes^1,2^; acting as a sponge to titrate away microRNAs^3–5^; or base-pairing with mRNAs as a way of regulating mRNA stability^6^. Defining these mechanisms requires identifying lncRNA functional domains and relevant interacting proteins. While mass spectrometry-based technologies have enabled the latter^7,8^, methods to systematically map lncRNA functional domains remain lacking.

The CRISPR (Clustered Regularly Interspaced Short Palindromic Repeats)-Cas9 (CRISPR associated protein 9) system can target specific genomic loci using single guide RNAs (sgRNA) and generate InDel (insertion or deletion) mutations^9,10^. For protein-coding genes, InDels often produce frame-shifts that give rise to truncated proteins^11,12^. However, for genes encoding lncRNAs, we reasoned that gene function would be perturbed only when InDels occur within a functional lncRNA domains and that such an association could be exploited to systematically screen for those regions. Thus, we asked if CRISPR technology could identify functional domains of the lncRNA X-inactive specific transcript (Xist).

Xist, a 17 Kb lncRNA, has served as a flagship model to study lncRNA functions. Since its discovery in early 1990s, extensive work has shown that endogenous or ectopically expressed Xist epigenetically silences genes or entire chromosomes, such as the X chromosome in female cells^13–15^. Upon expression, Xist “coats” the chromosome from which it is transcribed, and its spread recruits silencing factors, such as Polycomb proteins^2,16,17^, to transcriptionally inactivate gene expression *in cis*. Based on sequence conservation across species, several regions of Xist are known to be functionally important^18–21^, among them, a repeat region at the 5′ end, termed the A-repeats, which is required for Xist-mediated gene silencing^21^. Applying Tiling CRISPR, we mapped a Xist silencing domain to A-Repeats, suggesting the method’s feasibility. Furthermore, in the course of that analysis, we discovered that Xist A-repeats can promote Xist transcription.

## Results

### Tiling CRISPR identifies a 2.4 Kb region at the Xist 5′-end as a potential silencing domain

**Figure 1:**
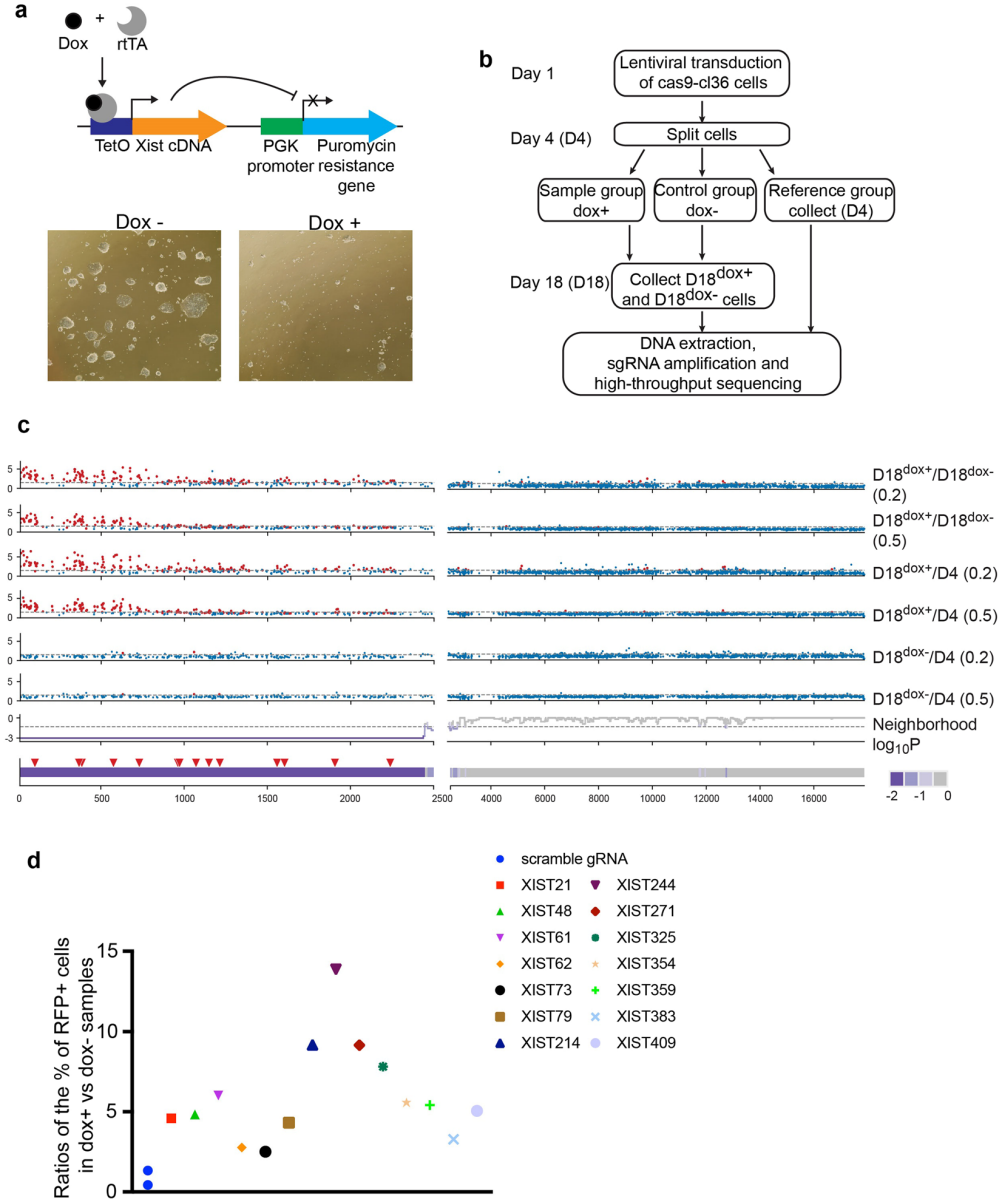
Tiling CRISPR identifies a 5′ region corresponding to a sgRNA cluster, as a potential Xist silencing domain. (**a**) Upper Panel: Schematic showing reporter system in which Xist induction is inversely correlated with expression of a puromycin resistance gene. Lower Panel: Representative images of Cl36 cells 4 days after culturing in puromycin-containing medium with or without 1 μg/ml doxycycline. Results indicate robust cell death following Xist induction. (**b**) Screening work flow. (**c**) Detection of enriched sgRNAs and enriched sgRNA clusters. The top 6 panels show enrichment profiles of individual sgRNAs among indicated samples. Dashed horizontal lines represent a FC level of 1.5. In panels 1–4, 197 sgRNAs that are significantly enriched with maxFC ≥ 1.5 and RSA p < 0.5 in D18^dox+^ samples are colored in red and the rest in blue. Among them, 3 sgRNAs are also enriched in D18^dox−^ samples (highlighted in red in panels 5–6). The 7^th^ panel shows neighborhood Log_10_P for each sgRNA within a sliding window. These values were used to identify an sgRNA-enriched cluster (see Methods). The bottom schematic shows a ~2.4 kb Xist region corresponding to an enriched sgRNA cluster, as determined by neighborhood Log_10_P. Red triangles represent 14 individual sgRNAs used for validation and downstream analysis. (**d**) Upon being transduced with an indicated sgRNA, cas9-cl36 cells were split into two groups with one group treated with doxycycline (dox+) and the other with DMSO (dox−). After 7 days continued culturing in puromycin, the ratio of the percentage of RFP+ cells in dox+ vs dox− samples were calculated.

To screen for Xist silencing domains, we employed a reporter line in which doxycycline (dox)-mediated Xist induction silences expression of a linked puromycin resistance reporter (puro^r^), a loss that would kill cells grown in puromycin-containing medium (Fig. 1a)^22^. This reporter line, known as cl36^22^, is ideal for our screen since: (i) it is derived from male embryonic stem cells in which endogenous Xist is expressed at low levels without known function to allow transgene analysis^22^; and (ii) cells harboring InDels that disrupt Xist silencing function would survive puromycin selection following Xist transgene induction, providing a positive selection for sgRNAs targeting that domain. To unbiasedly and comprehensively identify Xist silencing domains, we designed 1527 sgRNAs that target only Xist in the genome and tile the entire transgene (Supplementary Table 1), a method we designate “Tiling CRISPR”.

We cloned sgRNAs into an engineered lentiGuide-RFP (red fluorescent protein) vector. Upon confirmation of >90% sgRNA library coverage by high throughput-sequencing analysis, we infected cl36 reporter cells expressing cas9 (cas9-cl36) with 700 copies of each sgRNA to ensure its presence. To prevent large deletions due to the presence of >1 sgRNA/cell, we employed low Multiplicities Of Infection (MOI = 0.5 and 0.2). Four days later, we split cells into 3 groups: a sample dox−treated group (to induce Xist (dox+)); a Dimethyl sulfoxide (DMSO) -treated control group (dox−); and a reference group, which was immediately harvested (Fig. 1b). Sample and control groups were cultured for 14 more days in puromycin to allow proliferation of cells harboring desired InDels and enrichment of corresponding sgRNAs. Upon harvesting, we designated the experimental group as D18^d^°^x+^, the control group as D18^dox−^, and the reference group as D4. We then extracted genomic DNA from each sample for sgRNA amplification and high-throughput sequencing to identify enriched sgRNAs. Fold-changes (FCs) for each sgRNA were then quantified using normalized RPMs in sample vs. control groups (D18^dox+^/D18^dox−^ at a MOI of 0.2 or 0.5), sample vs reference groups (D18^dox+^/D4 at a MOI of 0.2 or 0.5), or control vs reference groups (D18^dox−^/D4 at a MOI of 0.2 or 0.5). To identify enriched sgRNA hits from 4 D18^dox+^ samples (D18^dox+^/D18^dox−^, 0.2 MOI; D18^dox+^/D18^dox−^, 0.5 MOI; D18^dox+^/D4, 0.2 MOI; and D18^dox+^/D4, 0.5 MOI), we used Redundant siRNA Analysis (RSA)^23^ for statistical analysis and assigned the maximum FC (maxFC) among the 4 FCs to each sgRNA. We identified 197 sgRNAs as hits based on RSA P ≤ 0.05 and maxFC ≥ 1.5. Among them, 3 showed a FC ≥ 1.5 in control groups (D18^dox−^/D4 at MOI 0.2 and 0.5). Thus, we detected 194 enriched sgRNAs in total (supplementary Table 1).

We reasoned that similar phenotypes would arise from mutations generated by adjacent or overlapping sgRNAs; thus, a “sgRNA cluster” would likely correspond to a true functional domain. Applying “sliding window” analysis with a window size of 30–300 bp, we identified one sgRNA cluster corresponding to the 15 to 2446 bp candidate region at the Xist transgene 5′-end (Fig. 1c, 2 bottom panels). Among 295 sgRNAs derived from this region, 167 were enriched, representing a hit rate of 56.6%, which is significantly higher than the overall hit rate of 12.9% when considering the entire transgene (p-value = 1.06e-107). We then randomly picked 14 enriched sgRNAs located across the candidate region for validation (Fig. 1c, bottom panel, red triangles). Upon transduction of individual sgRNAs, we determined sgRNA enrichment by measuring ratios of RFP+ cells between dox+ and dox− cells after 7 days of culture in puromycin. In comparison to scrambled sgRNA controls, which displayed ratios < 1, candidate sgRNAs displayed ratios ranging from 2.5 to 13.8 (Fig. 1d), confirming their enrichment. Overall, results derived from Tiling CRISPR suggest that a region at the Xist 5′ end is responsible for silencing function. Indeed, this region contains several conserved repeats that reportedly regulate Xist activity^18–21^.

### PacBio-seq suggests A-repeats within the 2.4 Kb region as the silencing domain

**Figure 2:**
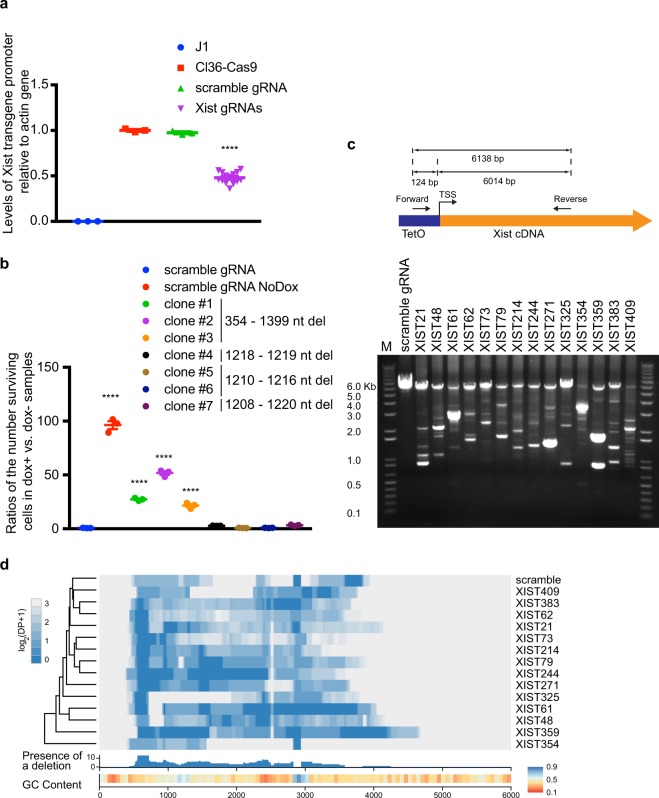
PacBio-seq suggests A-repeats within the 2.4 Kb region as a silencing domain. (**a**) Cas9-cl36 cells were transduced with scrambled or Xist-derived sgRNAs, followed by Xist induction and puromycin selection for 7 days. Xist transgenes containing intact promoters were quantified using qPCR of genomic DNA isolated from indicated groups. PCR reactions using J1, the parental line of cl36 cells, served as a negative control for primer specificity as this line harbors no Xist transgene, and no PCR products should be present. J1: n = 3; Cl36-Cas9: n = 3; scramble gRNA: n = 3; Xist gRNAs: n = 51. Graphs show means ± SEM. *P* values were generated by one-way ANOVA (*n* ≥ 3) followed by Bonferroni’s *post hoc* test. ****P < 0.0001. (**b**) For each indicated clone, cells were split into two groups with one group treated with doxycycline (dox+ ) and the other with DMSO (dox−). After 4 days continued culturing in puromycin, the ratio of the number of surviving cells in dox+ vs. dox− samples were calculated. Graphs show means ± SEM. *P* values were generated by one-way ANOVA (*n* = 3) followed by Bonferroni’s *post hoc* test. ****P < 0.0001. (**c**) Ethidium bromide-stained agarose gel showing PCR products amplified from a 6138 bp region of the transgene using genomic DNA derived from cas9-cl36 transduced with indicated sgRNAs. (**d**) Detection of common InDels on a Xist transgene generated by each of the 14 sgRNAs based on PacBio long-read sequencing. The heatmap shows coverage of depth (DP) profiles using DP^norm^ (see Methods) that represent coverage of sequencing depth of each nucleotide from samples and scrambled control. Below the heatmap, deletion results are summarized within 14 samples. Presence of a deletion is defined as the number of samples that harbor a deletion per position. Since GC content of a genomic region can impact sequencing depth, we also show GC content computed for 100 bins of 60 bp throughout the 6 kb region of the Xist 5′-end (bottom panel).

To narrow down silencing sequences within this region, we analyzed InDels generated by each of the 14 validated sgRNAs. We first determined internal vs. promoter InDels, as either would interfere with Xist function, while only internal InDels were applicable to domain analysis. We compared levels of a ~100 bp amplicon covering the Xist transgene transcription start site (TSS) in transduced vs. parental cas9-cl36 cells using qPCR analysis (Supplementary Table 2). Relative to the parental cas9-cl36 control, 36–58% cells infected with Xist-derived sgRNAs displayed intact promoters (Fig. 2a), suggesting ~ half of InDels are likely internal that do not perturb promoter function. The CRISPR-cas9 system generates both large and small Indels. To assess which types are likely responsible for loss of Xist silencing function, we derived cas9-cl36 clones infected with sgRNA Xist325 derived from a 20 bp region 1213 bp downstream of Xist transgene TSS and outside conserved repeats. Among 7 clones generated, 3 displayed 1046 bp deletions and the rest showed 2–13 bp deletions (Fig. 2b). For each clone, we calculated the proportion of surviving cells between dox+ and dox− cells after 4 days of culture in puromycin. Proportions ranging from 22–52% were detected from clones containing >1 kb deletions (Fig. 2b), in comparison to <5% from clones with small InDels. Therefore, we focused further analysis on large InDel detection.

A 6138 bp region at the Xist 5′-end was PCR-amplified using genomic DNA extracted from RFP+ cas9-cl36 cells that had been transduced with one of the 14 sgRNAs and cultured in dox plus puromycin for 7 days (Fig. 2c). To exclude InDels from the endogenous Xist gene, we used an upstream primer located at the Xist transgene promoter plus a downstream internal primer (Supplementary Table 2). Analysis of PCR products suggested the presence of large deletions in all samples (Fig. 2c). We then used PacBio long-read sequencing to identify and align deletions. While each sample displayed unique deletion patterns, a “common” deletion from 553 to 718 bp was detected in 13 of 14 samples, but not in the scrambled control (Fig. 2d), suggesting that this region, which is located within the Xist A-repeats, is essential for silencing function.

### A-repeats are required for Xist transactivation

**Figure 3:**
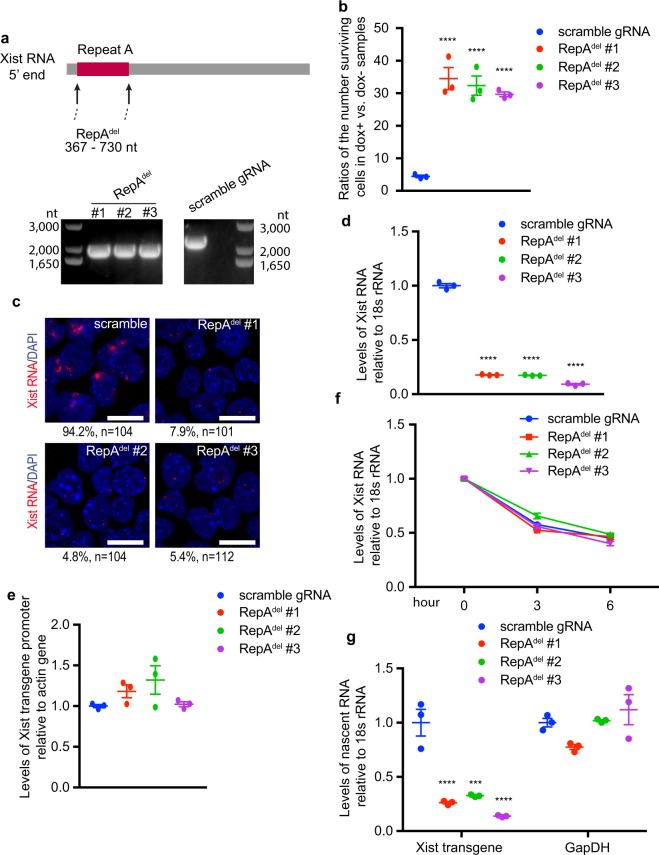
A-repeats positively regulate Xist transcription. (**a**) Upper Panel: Schematic showing positions of A-repeats (RepA^Del^). Lower panel: Genotyping results demonstrating deletion of A-repeats in the clones used for downstream analysis. (**b**) For each indicated clone, cells were split into two groups with one group treated with doxycycline (dox+) and the other with DMSO (dox−). After 4 days continued culturing in puromycin, the ratio of the number of surviving cells in dox+ vs. dox− samples were calculated. Graphs show means ± SEM. *P* values were generated by one-way ANOVA (*n* = 3) followed by Bonferroni’s *post hoc* test. ****P < 0.0001. (**c**) Representative Xist RNA FISH showing lack of Xist clouds in RepA^del^ clones. The percentage of nuclei containing Xist clouds is indicated. Scale bar = 20 μM. (**d**) RT-qPCR analysis of Xist RNA levels 24 hours after dox induction. Graphs show means ± SEM. *P* values were generated by one-way ANOVA (*n* = 3) followed by Bonferroni’s *post hoc* test. ****P < 0.0001. (**e**) Quantitative PCR indicating lack of promoter deletions in RepA^del^ clones. Graphs show means ± SEM. PCR primers and experimental conditions are the same as those used to generate Fig. 2a. (**f**) RT-qPCR to detect induced Xist RNA levels from indicated cells treated with Actinomycin D for 0, 3, or 6 hrs. Graphs show means ± SEM. *P* values were generated by two-way ANOVA (*n* = 3) followed by Bonferroni’s *post hoc* test. (**g**) Determination of nascent Xist RNA levels after 30 min of BrU labeling, followed by RNA pull-down with anti-BrU antibody. GapDH levels served as negative control. Graphs show means ± SEM. *P* values are generated by one-way ANOVA (*n* = 3) followed by Bonferroni’s *post hoc* test. ***P < 0.001, ****P < 0.0001.

To assess this potential function, we derived clones in which the entire A-repeat region (367–730 bp) had been deleted using CRISPR-cas9-directed homologous directed recombination (HDR) (Fig. 3a, upper panel, and Supplementary Table 2; clones are designated RepA^Del^). Following clone screening, we randomly picked 3 RepA^del^ clones for analysis (Fig. 3a, lower panel, and Sanger sequencing). We first assessed cell survival upon Xist induction in puromycin. Unlike the scrambled control which displayed ~5% cell survival, 30–35% of cells from RepA^del^ clones survived (Fig. 3b), confirming that A-repeats function in *puro*^*r*^ silencing. To assess underlying mechanisms, we evaluated Xist levels by RNA FISH and RT-qPCR following induction. Both methods revealed a significant lack of Xist RNA in RepA^del^ clones (Fig. 3c,d). After excluding the possibility of promoter deletion in all clones (Fig. 3e), we reasoned that loss of the A-repeats may either inhibit Xist transcription or promote Xist decay. To determine which, we evaluated Xist half-life by actinomycin D treatment and detected no changes in RepA^del^ clones vs controls (Fig. 3f), suggesting decreased Xist RNA levels detected from RepA^del^ clones are not due to enhanced Xist RNA decay. We then evaluated Xist RNA synthesis in all clones by measuring nascent Xist RNA levels after 30 min of bromouridine (BrU) labeling and immunoprecipitation of labeled RNA. Relative to scrambled controls, RepA^del^ clones displayed a >67% decrease in levels of nascent Xist RNA (Fig. 3g), while no change was detected from nascent GapDH mRNA, suggesting that loss of A-repeats downregulates Xist transcription. Overall, these experiments suggest that the RepA region is required for Xist transactivation.

## Discussion

Although lncRNAs have been extensively analyzed, tools useful to assess their function are limited and mostly borrowed from methods initially devised to define mRNA activity. For example, lncRNA loss-of-function studies have been based on use of RNA interference to degrade lncRNA^24,25^ or on the CRISPR-Cas9 to either repress lncRNA expression through promoter manipulation^26^ or to generate large deletions of the lncRNA gene loci^27^. These technologies have been effective in identifying biologically relevant lncRNAs, but it has remained difficult to push lncRNA functional analysis forward. Currently, lncRNA functional domains are often predicted based on RNA sequence conservation. However, it is well-established that functionally conserved lncRNAs show poor sequence conservation^28,29^, greatly limiting the utility of these approaches. In contrast, the technology we developed, Tiling CRISPR, directly identifies lncRNA functional sequences, whether they are highly or poorly conserved, in an unbiased manner. In addition, if multiple lncRNAs function in the same molecular pathway, it is possible to screen domains of multiple lncRNAs at the same time – thus, we envision Tiling CRISPR is also a method amendable to high-throughput screen.

In this proof-of-concept study, using Xist lncRNA as a model, we demonstrated the feasibility of Tiling CRISPR. Our design of tiled sgRNAs was based on several considerations: 1) Like shRNAs, not all sgRNAs are effective in generating InDels. Since the only requirement for sgRNA design is that target sites are immediately followed by a Photospacer Adjacent Motif (PAM, 5′-NGG-3′), then by chance, every 8-nucleotide on either the forward or reverse strand would contain a PAM sequence and could be targeted by sgRNA. Such high coverage greatly increases the efficiency of mutation generation. 2) Off-target effects of individual sgRNAs are well documented^30–33^. When applying Tiling CRISPR, we observed that functional sgRNA forms clusters, i.e. multiple functional sgRNAs are enriched at certain loci (Fig. 1c). Cluster formation suggests that mutations associated with neighboring sgRNAs give rise to similar phenotypes, greatly reducing concerns relevant to sgRNA off-target effects. We also observed that unlike traditional CRISPR studies, in which InDels are predominantly small, we detected large deletions in our screen (Fig. 2c,d). Indeed, use of the Non-Homologous End Joining (NHEJ) CRISPR system, in which Cas9 and a single sgRNAs are introduced into cells without a donor sequence to direct homology end repair, reportedly generates a spectrum of genomic InDels, with the largest deletion up to 6 Kb^34^. Since small Indels are unlikely to abolish lncRNA function, we envision that Tiling CRISPR will primarily detect large deletions, as evidenced by Xist analysis.

Using Tiling CRISPR, we successfully identified the known Xist silencing domain, A-repeats. A-repeats reportedly mediate silencing through multiple mechanisms: interacting with silencing factors^2,35^, recruiting genes into a Xist-mediated silencing compartment^36^, regulating Xist spreading^37,38^, or regulating Xist splicing^39^. However, our findings suggest a novel function whereby A-repeats positively regulate Xist transcription. In agreement, a genetic study has reported lack of Xist RNA and failure of X-inactivation when deleting A-repeats in mouse female embryos^40^. In addition to A-repeats, two other repetitive sequences, including F and C repeats located at the Xist 5′-end downstream of the A-Repeats, reportedly regulate Xist spreading^41–43^. We did not detect these regions using Tiling CRISPR, possibly because their loss has relatively subtle effects on Xist-mediated gene silencing compared to A-repeats deletion. This idea is supported by a previous study showing that deletion of Repeats F or C alone did not alter Xist-mediated puro^r^ silencing^21^.

Overall, we conclude that Tiling CRISPR provides a new tool to map lncRNA functional domains in an unbiased and potentially high-throughput manner. Domain identification will advance lncRNA research by enabling in-depth mechanistic analysis of lncRNA activity and will enable development of RNA-based therapeutics, such as oligonucleotides, useful to effectively target lncRNAs and block their activity in disease.

## Materials and Methods

### Cell culture

Cl36 mouse embryonic stemm cells (mESCs) were cultured on 0.2% gelatin (Sigma) coated dishes at 37 °C with 5% CO_2_ in ESC medium: DMEM (Gibco) supplemented with 15% fetal bovine serum (FBS, Gibco), 25 mM HEPES (Gibco), 2 mM glutaMAX (Gibco), 0.1 mM non-essential amino acids (Gibco), 0.1 mM β-mercaptoethanol (Sigma), 500 units/ml leukemia inhibiting factor (LIF, Millipore), 3 μM CHIR99021 (Sigma), 1 μM PD0325901 (Sigma), and 2 μg/ml penicillin-streptomycin (Gibco). For Xist induction, 1 μg/ml doxycycline were added to the medium. For selection, 2 μg/ml puromycin (Millipore) were added.

To express cas9 in Cl36, cells were transduced with lentivirus containing EF1α-3 × FLAG-NLS-Cas9-T2A-bsd(R). 10 μg/ml blasticidin (Millipore) was added into culture medium for 3 days to select cells expressing cas9.

### Tiling single guide RNA (sgRNA) design and cloning

The Xist sequence was adopted from RefSeq entry NR_001463.3 with chromosome range chrX:103460373-103483233 on GRCm38. sgRNAs are designed following these rules: (i) they are 20 bp long; (ii) target sites are immediately followed by 5′-NGG PAM (Photospacer Adjacent Motif), a motif required for Cas9 endonuclease activity^44–46^; (iii) and sgRNAs originate from both forward and reverse strands of Xist cDNA. A total of 1660 unique sgRNA sequences were identified from both strands with an average separation of 12.7 bp. After removing sgRNAs that match multiple locations on the mouse genome, 1527 sgRNAs were retained. The Rule Set2^47^ on-target scores for the sgRNAs vary with values of 0.48 ± 0.13. Retained sgRNAs were synthesized by Custom array Inc., amplified by PCR, and cloned into the *Bbs*I restriction sites of lentiviral U6 sgRNA expression vector (lentiGuide-RFP).

### CRISPR library screen

Cl36-Cas9 cells were transduced with lentiviral sgRNA pool at a low multiplicity of infection (MOI = 0.2 or 0.5) and a representation of 700 cells per sgRNA. 2 × 10^6^ (MOI = 0.5) or 5 × 10^6^ (MOI = 0.2) Cl36-Cas9 cells were seeded in 15-cm 0.2% gelatin coated dishes at a density of 1 × 10^6^ cells/dish in ESC medium containing 10 μg/μl polybrene and lentivirus. Cell culture medium was changed after overnight incubation. Four days after transduction, cells were divided into 3 groups with cells from reference groups harvested and sample or control groups cultured in puromycin containing medium with or without 1 μg/ml doxycycline (Sigma), respectively. Survived cells were collected 14 days after culture. The groups are designated as D18^dox+^, D18^dox−^, and D4. Genomic DNA was extracted using DNeasy® Blood & Tissue Kit (QIAGEN) according to the manufacturer’s instructions. The sgRNA cassettes were PCR amplified and sequenced with standard Illumina Hiseq. 1000 and protocols.

### sgRNA primary hit identification

Reads were mapped to Xist sequence using BWA v0.5.9^48^ and the aligned reads were counted by SAMTools v0.1.18^49^. Quantile normalization was performed to reads per million base (RPM) to remove bulk difference across samples. Fold changes (FCs) at sgRNA level were then computed using the normalized RPMs between sample vs. control groups (D18^dox+^/D18^dox−^ at MOI 0.2 or 0.5), sample vs reference groups (D18^dox+^/D4 at MOI 0.2 or 0.5), or control vs reference groups (D18^dox−^/D4 at MOI 0.2 or 0.5). To identify enriched sgRNA hits from 4 D18^dox+^ samples (D18^dox+^/D18^dox−^ 0.2, D18^dox+^/D18^dox−^ 0.5, D18^dox+^/D4 0.2 and D18^dox+^/D4 0.5), we used Redundant siRNA Analysis (RSA)^23^ for statistical analysis and assigned maximum FC (maxFC) among the 4 FCs to each sgRNA. 197 sgRNAs were identified as hits based on RSA P ≤ 0.05 and maximum FC ≥ 1.5. Among them, 3 sgRNAs showed FC ≥ 1.5 in control groups (D18^dox−^/D4 at MOI 0.2 and 0.5). Thus, we detected 194 enriched sgRNAs in total.

### sgRNA cluster detection

Centered at each position, the distribution of FC values formed by its nearby sgRNAs within the ±*n*-bp window was compared to value 1 using one sample t-test, where *n* ranges from 15 to 150 with an increment of 1 to scan for the optimal window size resulting in the lowest p-value (P_Ttest). To correct for multiple-test effect, all FC values were randomly shuffled and the whole search process were repeated 1000 times to simulate the NULL distribution. The permutation test assigned each position a new P_perm defined as the number of simulations with p ≤ P_Ttest divided by 1000. P_perm was further smoothed by expanding P_perm to positions within the same optimal window; then the minimum P_perm at each position were defined as its P_smooth. All p-values were calculated independently for each of the 4 experimental groups (D18^dox+^/D18^dox−^ at MOI 0.2 or 0.5 or D18^dox+^/D4 at MOI 0.2 or 0.5) independently for each of the 4 D18^dox+^ related FCs, which results in 4 sets of p values per position. At each position, the least significant P_smooth across all FCs was considered as the neighborhood P-value (Fig. 1c). An sgRNA cluster is defined as a region containing sgRNAs that display neighborhood P ≤ 0.01.

### Individual sgRNA validation

14 sgRNA hits were randomly selected from 100 to 2250 bases relative to the 5′ end for validation. Individual sgRNA were cloned into lentiGuide-RFP vector and were used in lentiviral packaging and infection. Cas9-cl36 cells were transduced at MOI of 0.1with lentivirus containing scramble sgRNA or individual Xist sgRNA. 4 days post-transduction, cells were divided into puromycin-containing ESC medium and were treated with either 1 μg/ml doxycycline (dox+) or DMSO (dox−). 7 days later, the ratio of RFP+ cells in dox+ vs. dox− treatments were calculated.

### PacBio single molecule, real-time (SMRT) sequencing

Cas9-cl36 were transduced with virus containing a scrambled sgRNA or 14 Xist-derived sgRNAs used for validation. For scrambled sgRNA control, RFP+ cells were FACS sorted without dox/puro selection. For 14 Xist-derived sgRNAs, RFP+ cells were FACS sorted 7 days after dox/puromycin selection. Genomic DNA from these cells were extracted. A ~6 Kb target region located at Xist 5′-end (Supplementary Fig. 1e and Supplementary Table 2) was amplified using barcoded primers and PrimeSTAR GXL DNA Polymerase (Takara Bio), purified with AMPure PB beads (Pacific Biosciences) and sequenced on a PacBio Sequel sequencing platform (Pacific Biosciences, RTL Genomics). Circular consensus (CCS) reads were obtained from standard Pacbio sequencing analysis pipeline using at least 3 subreads from the same circularized single DNA molecule. All CCS reads were aligned to Xist sequence using Blasr^50^ with 99.9% identity (minPctIdentity = 99.9) and the average mapping rate was 42%.

### Deletion detection

After removing PCR duplicates using SMRT Tools, SAMTools^49^ was applied to compute depth of coverage (DP) for each position. The average DP ranged from 135 to 1853 for sgRNA samples and was 20.3 for the scramble control. In order to correct for background DP variation across samples, DP at position *i* was normalized by the median of background DP at logarithmic scale for each sample:

$${D}{{P}}_{{i}}^{{norm}}=\frac{{\mathrm{log}}_{2}({D}{{P}}_{{i}}+1)}{{median}({\mathrm{log}}_{2}({D}{{P}}_{{b}}+1))}$$

where *b* represents positions in the background region without InDel mutations. According to DP profiles, 5 kb–6 kb region was selected as the normal background. A position *i* was defined as deletion if ${D}{{P}}_{{i}}^{{norm}}\le 0.1$. In order to identify sgRNA-induced deletions, all deletion positions of the scramble sample were excluded. The most frequent deletion (553 to 718 bp) were shared by 13 out of 14 sgRNA samples

### CRISPR-Cas9 mediated homologous directed recombination

To precisely deleting A repeats and the region after A repeats, Cas9-Cl36 cells were transfected with donor plasmid with homologous arms cloned in pUC19 and 2 lentiGuide-RFP constructs containing a pair of sgRNA flanking the target region. 72 hours after transfection, RFP positive cells were sorted (BD FACSAria II) and seeded into 96-well plates at 1 cell per well. Individual clones were expanded and genotyped by PCR and Sanger sequencing.

### Cell viability assay

Cells were seeded into 96-well plate at 5,000 cells per well and cultured in puromycin containing medium with or without 1 μg/mL doxycycline for 4 days. Cell viability was determined with CellTiter-Glo^®^ Luminescent Cell Viability Assay (Promega) using CLARIOstar^®^ microplate reader (BMG LABTECH).

### XIST RNA FISH

FISH experiment was carried out as previously described^2^. Xist expression was induced in the puromycin free ESC medium containing 1 μg/ml doxycycline for 48 hours and ES cells were dissociated and collected by cytospin. The slides were treated by CSK with 0.5% triton prior to paraformaldehyde fixtion. Xist pSx9-3 probe was labeled with Cy3-dUTP by nick-translation (Roche).

### Assessment of Xist RNA stability

Xist expression was induced in the puromycin free ESC medium containing 1 μg/ml doxycycline for 24 hours. To assess RNA stability, actinomycin D (Sigma) at 5 μg/ml was added to cell culture and after 0, 3 or 6 hrs of incubation, cells were collected and RNAs were isolated for RT-qPCR.

### Assessment of Xist RNA synthesis

Experimental procedure is adopted from a previous publication^51^. Briefly, 2 mM Bromouridine (BrU, Sigma) was added to medium and cells were incubated with BrU at 37 °C for 30 min. RNAs were isolated and BrU containing RNAs were pulled down for RT-qPCR using anti-BrU antibody (BD bioscience, Cat. # 555627) and Protein A/G beads (Thermo Fisher Scientific, Cat. # 88802).

### Life Sciences Reporting Summary

Further information on experimental design and reagents is available in the Life Sciences Reporting Summary.

## Electronic supplementary material

Supplementary Table 1

Supplementary Table 2

## Data Availability

All high throughput seq data were deposited to Sequence Read Archive (SRA) under BioProject ID PRJNA507802. The remaining data that support the findings of this study are available from the corresponding author upon reasonable request.
